# Plant Signaling, Behavior and Communication

**DOI:** 10.3390/plants13081132

**Published:** 2024-04-18

**Authors:** Frantisek Baluska, Gustavo Maia Souza

**Affiliations:** 1 Institute of Cellular and Molecular Botany (IZMB), University of Bonn, 53115 Bonn, Germany; 2 Department of Botany, Institute of Biology, Federal University of Pelotas, Pelotas 96160, RS, Brazil

Being sessile organisms that need to effectively explore space (above and below ground) and acquire resources through growth, plants must simultaneously consider multiple possibilities and wisely balance the energy they spend on growth with the benefits for survival. Unlike animals whose body structure is set early in their development, plants have a modular architecture that grows indefinitely from meristems, which are versatile tissues found throughout the plant, particularly at shoot and root apexes. This growth results in new branches, shoots, leaves, and roots throughout a plant’s life, facilitating resource acquisition. Additionally, these modules are more than mere building blocks; they allow plants to perceive and locally react to environmental stimuli, thus enabling a range of appropriate responses to different signals and threats. This modularity is not indicative of isolated units but rather a cohesive network that allows for self-organization across multiple scales—from molecular to cellular levels and from individual organisms to entire communities—creating a complex, integrated signaling system within the plant [1,2].

Emerging areas in plant sciences are increasingly focusing on plant signaling, communication, cognition, and behavior. New research has shown that plants are far more intricate and engaged in their interactions with both living and non-living environments. This Special Issue was focused on the unique sensory systems of plants, including the detection and transmission of signals, the gathering and processing of sensory information related to actively adapting to stress, and the dynamics of communication among plants and their surroundings, including other plants and other living beings. Therefore, it is not surprising that a large amount of evidence has been accumulated showcasing astonishing cognitive plant abilities, such as their ability to accurately find resources, to make decisions, and to communicate with each other about their “findings”.

Herein, the exquisite dynamic behavioral capacities of plants that are embedded in an ever-changing environment are well illustrated by Pavlovic et al. [3], evidencing the accurate and selective way by which the carnivorous plant *Drosera capensis* senses and behaves with regard to different stimuli. Costa et al. [4] document the abilities of plants to respond to different cues that, presented as single or combined stimuli, engender different systemic signals (both electrical and hydraulic). The changes in the dynamic of different systemic signals, particularly the bioelectrical ones, induce different responses at modules distant from the local stimulated tissues, highlighting plants’ capacities to integrate and to coordinate responses systemically. Such ability to integrate signals is also demonstrated at the tissue level when collective stomatal behavior changes under different external cues (light and drought), affecting photosynthetic efficiencies—as showed in the mathematical simulations by Sukhova et al. [5]. Exemplifying the plethora of sophisticated mechanisms used by plants to sense their environment, Yamashita and Baluska [6] propose that plant eye-like ocelli, which allow plant-specific kind of vision [7,8] and which evolved from the algal ocelloids, are part of complex plant sensory systems and guide cognition-based plant behavior, such as the mimicking of diverse host plants by woody vine *Boquila trifoliata* [9,10,11] and root light escape tropism [12].

How plants sense the environment and then behave is not only a matter of perceiving and processing different external cues. The area where plants are integrated can also affect their ability to explore available resources. Mahal at al. [13] showed the ability of wheat plants to explore nutrients, varying both in their amount and distribution in the soil, exhibiting not only belowground responses but also aboveground changes; however, in this case, the influence of the area in which the plants grew was not clear. On the other hand, Chautá and Kessler [14] discussed how plants respond to changes in light quality and exposure to chemicals released by neighboring plants (volatile organic compounds, VOCs). The study found that these factors strongly interact and influence on the production of secondary metabolites, both volatile and non-volatile, in plants, affecting how plants detect and respond to VOCs emitted by other plants. The findings indicate that plants can integrate various environmental cues to modulate their chemical outputs, which in turn can affect the interactions within plant populations and communities.

Moreover, the ability of plants to communicate “stress calls” to other ones is well illustrated by Falik and Novoplansky [15], who report that drought cuing and relayed cuing is observed in intra- and interspecific neighbor combinations, but their strength depends on plant identity and position. Accordingly, Midzi et al. [16] highlight the ecological relevance of such interactions under various environmental stresses and the growing understanding of the mechanisms involved and the significance of VOC-mediated inter-plant interactions under both biotic and abiotic stresses. As an interesting example of inter-plant interactions, Le Ding et al. [17] show that intraspecific kin recognition may facilitate cooperation between genetically related biotypes to compete with interspecific rice, offering many potential implications and applications in paddy systems.

Facing a constantly changing environment and, at the same time, interacting with other plant species through a sophisticated communication system may require that, at some point, plants are challenged to make choices to ensure survival. Lee et al. [18] bring such exciting possibilities, suggesting that decision making is especially relevant to the issue of plant intelligence as it is commonly taken to be characteristic of cognition. As a matter of evidence, Wang et al. [19] present the case of pea plants searching for support and likely making choices between different possibilities, showing distinct preference for their support. Interestingly, the dynamics of pea plants’ climbing movements show distinct kinematic traits allowing for automatic classification using machine learning methods, as illustrated by Wang et al. [20]. Making decisions is considered a high-level cognitive capacity of living beings, surpassing the abilities of sensing and responding. In a complex and demanding environment, it is likely that making choices would demand the ability to attend specific cues more relevant for plant survival. In this direction, Parise et al. [21] have proposed that plants can show states of attention when facing specific challenges co-occurring with different cues. They suggest that the phenomenon of attention in plants would be reflected in their electrophysiological activity, which can be analyzed by investigating the potential existence of different band frequencies (including low, delta, theta, mu, alpha, beta, and gamma waves) using a protocol adapted from neuroscientific research.
